# Design and Analysis of a Compact Precision Positioning Platform Integrating Strain Gauges and the Piezoactuator

**DOI:** 10.3390/s120709697

**Published:** 2012-07-17

**Authors:** Hu Huang, Hongwei Zhao, Zhaojun Yang, Zunqiang Fan, Shunguang Wan, Chengli Shi, Zhichao Ma

**Affiliations:** College of Mechanical Science and Engineering, Jilin University, Changchun 130025, China; E-Mails: huanghuzy@163.com (H.H.); yzj@jlu.edu.cn (Z.Y.); fanzunq@jlu.edu.cn (Z.F.); wanshunguang@126.com (S.W.); shichengli163@163.com (C.S.); kobe2324@126.com (Z.M.)

**Keywords:** strain gauge, piezoactuator, precision positioning platform, calibration, closed-loop control

## Abstract

Miniaturization precision positioning platforms are needed for *in situ* nanomechanical test applications. This paper proposes a compact precision positioning platform integrating strain gauges and the piezoactuator. Effects of geometric parameters of two parallel plates on Von Mises stress distribution as well as static and dynamic characteristics of the platform were studied by the finite element method. Results of the calibration experiment indicate that the strain gauge sensor has good linearity and its sensitivity is about 0.0468 mV/μm. A closed-loop control system was established to solve the problem of nonlinearity of the platform. Experimental results demonstrate that for the displacement control process, both the displacement increasing portion and the decreasing portion have good linearity, verifying that the control system is available. The developed platform has a compact structure but can realize displacement measurement with the embedded strain gauges, which is useful for the closed-loop control and structure miniaturization of piezo devices. It has potential applications in nanoindentation and nanoscratch tests, especially in the field of *in situ* nanomechanical testing which requires compact structures.

## 1. Introduction

Taking advantages of high stiffness, compact size, unlimited displacement resolution, large force generation, fast response as well as low power consumption, piezoactuators have been widely used in the fields of precision positioning [1–3], nanofabrication [4–7], micromanipulation [8–10], scanning probe microscopes [11,12] and nanomechanical testing systems [13–16].

For different applications, various kinds of piezo driving platforms have been proposed by previous researchers. One important category among them is the actuator that integrates displacement sensors to realize high precision positioning via closed-loop control. Zhu [4], Kim [6,7] and Tian [17,18] designed the fast tool servo to realize precision turning or grinding by integrating laser or capacitive displacement sensors and piezoactuators. Shimamoto [14] proposed a nanoindentation tester by integrating the piezoactuator and an optical fiber displacement sensor. Park [13] also presented a precision indentation and scratching system by integrating the piezoactuator and a capacitive displacement sensor. Shiou [19] developed a real-time control closed-loop micro-/nano-positioning system by integrating a capacitive displacement sensor to overcome the problem of the hysteresis and nonlinearity of the piezoactuator and to increase the positioning speed of the positioning stage. The works mentioned above realize different applications by integrating the piezoactuator and commercial displacement sensors. Though accuracy or resolution of these sensors is high, dimensions are a little big which makes the size of the platforms large. For some special applications requiring limited dimensions, such as *in situ* nanomechanical tests inside the scanning electron microscope, applications of commercial displacement sensors are limited. In addition, complex mechanical assembly leads to stiffness degradation and decreased platform sensitivity. Also measurement errors arise from the installation of sensors or quality of measuring surface, so more compact structures and direct measuring methods should be developed.

Fleming [20] presented a new technique of strain and force feedback scheme for reduction of creep, hysteresis and vibration in piezoelectric actuated systems. The proposed scheme can be integrated into the piezoactuator which minimizes parts count and overall system cost. In [21], a piezoelectric strain sensor was bonded to a flexure-based nanopositioner as a displacement sensor for damping and tracking control. With damping and integral tracking control, a closed-loop bandwidth of 1.86 kHz was achieved. AFM imaging results demonstrate the efficacy of using a piezoelectric strain sensor for damping and tracking control of high-speed nanopositioners.

In this paper, a compact precision positioning platform integrating strain gauges and a piezoactuator is designed for a future application of *in situ* nanoindentation inside the scanning electron microscope. Four strain gauges are glued at the root of two parallel elastic plates to measure the output displacement of the piezoactuator directly. The work principle was introduced and the mathematical model of the strain gauge sensor was established. Effects of geometric parameters of two parallel plates on Von Mises stress distribution as well as static and dynamic characteristics of the structure were studied by the finite element method. Results of the calibration experiment and output performance testing demonstrate that the developed precision positioning platform has good linearity and it can be integrated into an *in situ* nanoindentation device to realize *in situ* nanoindentation tests of materials inside a scanning electron microscope.

## 2. Configuration and the Work Principle

**Figure 1.**
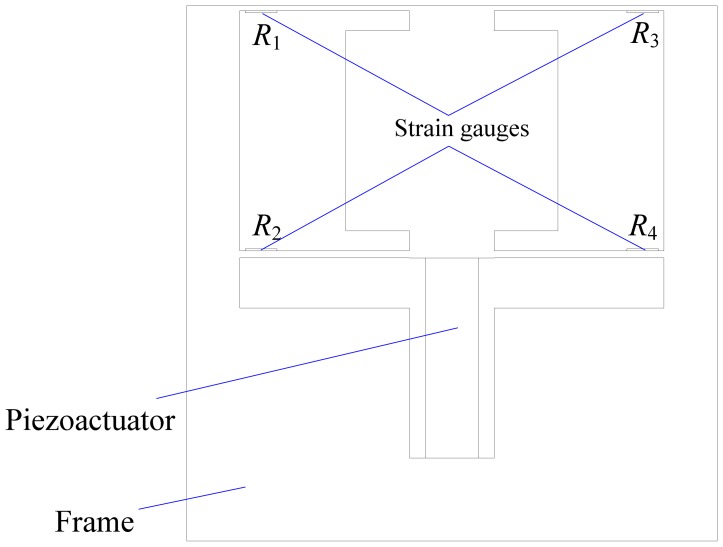
Schematic diagram of the developed platform.

Figure 1 is the schematic diagram of the developed platform, which mainly consists of a piezoactuator, four strain gauges and a flexure hinge frame. The piezoactuator is an AE0505D16F type piezoelectric stack (NEC Tokin) with maximum displacement of 17.4 ± 2.0 μm and a maximum generated force of 850 N. The strain gauges are BFC-350-3AA-11 type and the grid material is constantan with a resistance of 350 Ohm. The flexure hinge frame has two parallel elastic plates processed by wire cutting using the material 65 Mn and the strain gauges are adhered in the root of plates with M-Bond 610 adhesive. These two parallel plates, the piezoactuator and the frame form the positioning platform. Strain gauges and two parallel plates form the displacement sensor. The piezoactuator will extend when voltage signal is applied to it, and then pushes the two parallel plates to output precision displacement. At the same time, strain gauges will deform with deformation of the two parallel plates, causing their electrical resistance to change.

**Figure 2.**
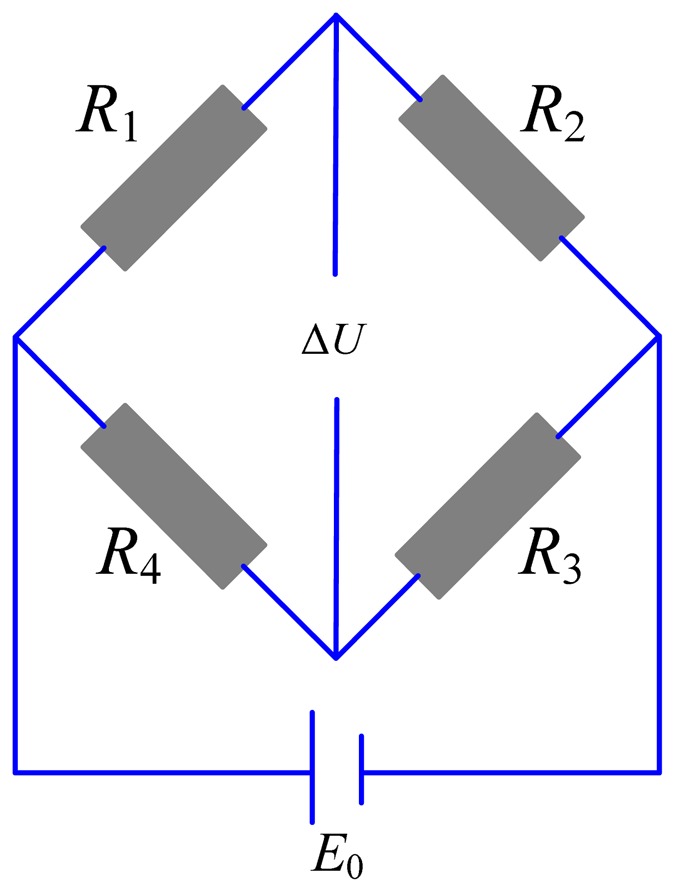
Wheatstone bridge.

The resistance change is converted to voltage change using the Wheatstone bridge as shown in Figure 2, which is conveniently measured by an A/D card, so the precision positioning platform is established. Compared with other platforms integrating commercial displacement sensors, such as the laser sensor or the capacitance sensor, the proposed platform can be designed with kinds of structures and also more compact because of the embedded strain gauges. The cost is also low.

## 3. The Mathematical Model of the Strain Gauge Sensor

**Figure 3.**
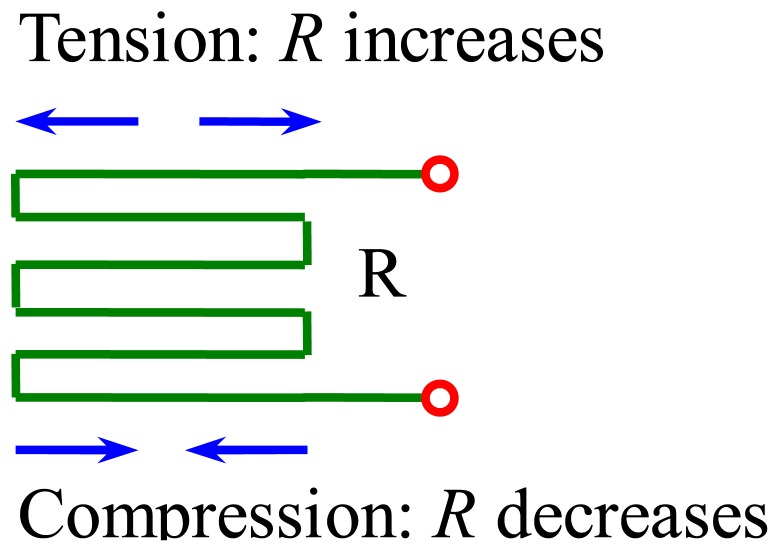
The principle of the strain gauge.

Figure 3 shows the principle of the strain gauge. The resistance *R* increases when tension is applied to it. In contrast, the resistance *R* decreases when it is compressed. Whether the force applied to the strain gauge is tension or compression, is decided by the installation location of the strain gauge.

**Figure 4.**
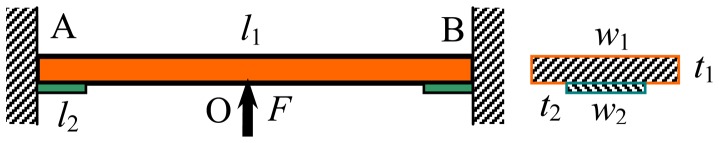
The simplified upper plate with strain gauges.

Now we will illustrate the strained condition of each strain gauge in detail. For purposes of analysis, the upper plate of the two parallel plates is simplified as shown in Figure 4. *F* is the driving force coming from the piezoactuator. *l*_1_, *w*_1_ and *t*_1_ are length, width and thickness of the elastic plate, respectively. *l*_2_, *w*_2_ and *t*_2_ are the length, width and thickness of the strain gauge, respectively. According to material mechanics, the location where strain gauges are installed in Figure 4 is tensioned, so resistance of these two strain gauges increases and the strain can be expressed by:

(1) $$ɛ=\frac{3Fl_{1}}{2Ew_{1}t_{1}^{2}}$$

where *E* is the elastic modulus of 65 Mn.

Similarly, resistance of strain gauges installed on the lower elastic plate decreases. Assuming that changes in resistance of strain gauges are Δ*R*_1_, Δ*R*_2_, Δ*R*_3_ and Δ*R*_4_ respectively, the voltage change in Figure 2 can be expressed by:

(2) $$\Delta U=\frac{E_{0}}{4}(\frac{\Delta R_{1}}{R_{1}}-\frac{\Delta R_{2}}{R_{2}}+\frac{\Delta R_{3}}{R_{3}}-\frac{\Delta R_{4}}{R_{4}})$$

The gauge factor *K*_i_ is defined as:

(3) $$K_{i}=\frac{\Delta R_{i}/R_{i}}{{ɛ}_{i}}(i=1,2,3,4)$$

where Δ*R_i_* is the change in resistance caused by strain; *R_i_* is the resistance of the undeformed strain gauge, and *ε_i_* is strain.

Considering that four strain gauges are the same and the installation location is symmetric, if the size of strain is *ε*, then:

(4) $${ɛ}_{1}={ɛ}_{3}=ɛ$$

(5) $${ɛ}_{2}={ɛ}_{4}=-ɛ$$

So Equation (2) can be simplified to:

(6) $$\Delta U=E_{0}Kɛ$$

where *K* is the gauge factor of the used strain gauges.

## 4. Mechanical Design and Analysis

According to Equations (1) and (6), the voltage change Δ*U* is proportional to strain *ε*, and geometric parameters of two parallel plates and strain gauges will affect the strain significantly. Usually, as a user, geometric parameters of the strain gauges cannot be changed, so the effects of the geometric parameters of the strain gauges on the strain are not analyzed in this paper. In this section, finite element analysis was carried out to study effects of geometric parameters of two parallel plates on Von Mises stress distribution as well as static and dynamic characteristics of the precision positioning platform.

### 4.1. Finite Element Analysis of Geometric Parameters of Two Parallel Plates

**Figure 5.**
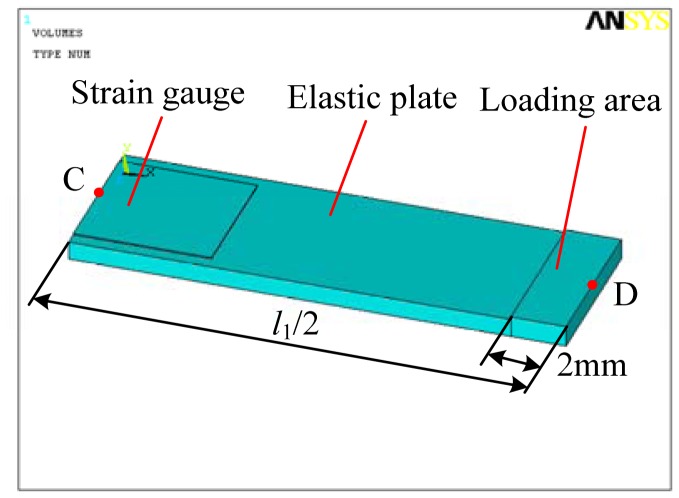
The simplified model of the strain gauge sensor.

Figure 5 is the simplified model of the strain gauge sensor, mainly consisting of the elastic plate, the strain gauge and the loading area. It is half of a single plate. Left middle point C and the right middle point D of the upper surface of the elastic plate are shown in this figure.

The strain gauge is glued on the upper surface while the loading area is added on the upper surface using Boolean operation of ANSYS 10.0 software. Elastic modulus and Poisson ratio of the elastic plate are 206 GPa and 0.288 respectively. While for the strain gauge, they are set to be 150 GPa and 0.3. Solid 95 is selected to mesh the model and key portions are refined to improve the simulation accuracy. On the left side of the elastic plate, all degrees of the freedom are constrained. The displacement load of 10 μm is applied on the loading area along the negative *y* direction. The symmetric boundary condition is applied on the right side. Basic parameters are set as follows: *l*_1_ = 36 mm, *l*_2_ = 4 mm, *w*_1_ = 6 mm, *w*_2_ = 5 mm, *t*_1_ = 0.7 mm, *t*_2_ = 0.05 mm. When analyzing effect of a single parameter of the elastic plate, other parameters are set to be the values of basic parameters.

Because the strain gauge is glued on the upper surface of the elastic plate, strain of the strain gauge can be thought as the same as that on the upper surface of the elastic plate. Accord to Hooke's Law of Elasticity, strain is proportional to stress. Through analyzing stress distribution of the elastic plate with different geometric parameters, as shown in Figure 4, the suitable installation location of the strain gauge can be selected and effects of these parameters on Von Mises stress distribution can be studied. In order to ensure that the plate has enough strength, stress distribution along the elastic plate is given.

**Figure 6.**
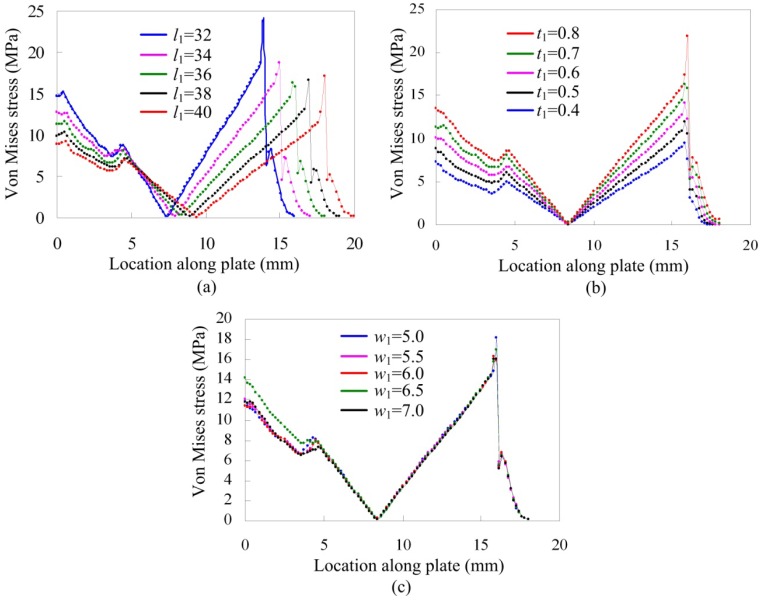
Von Mises stress distribution along the elastic plate with (**a**) different length *l*_1_; (**b**) different thickness *t*_1_; (**c**) different width *w*_1_.

Figure 6(a–c) are Von Mises stress distributions of the elastic plate from the left middle point C to the right middle point D of the upper surface with different length *l*_1_, different thickness *t*_1_ and different width *w*_1_, respectively. Von Mises stress increases when length *l*_1_ decreases, while stress of the middle part is very low and the lowest stress trends to 0 MPa for different length *l*_1_. Like the result of different length *l*_1_, Von Mises stress varies obviously with different thickness *t*_1_ and it increases with the increasing of thickness *t*_1_. Being different from length *l*_1_ and thickness *t*_1_, Von Mises stress is not sensitive to width change. So selection of length *l*_1_ and thickness *t*_1_ should be more careful.

Also, there are some common characteristics for these three parameters. Because of existence of the gauge, stress distribution has some variation though it is not sharp. Stress in the root of the plate or near the loading area is larger than other places, so strain gauges are suitable to be glued in the root of the plate or near the loading area. Due to space limit near the loading area where the piezoactuator is installed or stage existed, strain gauges were glued in the root of two parallel plates.

### 4.2. Static and Modal Analysis

**Figure 7.**
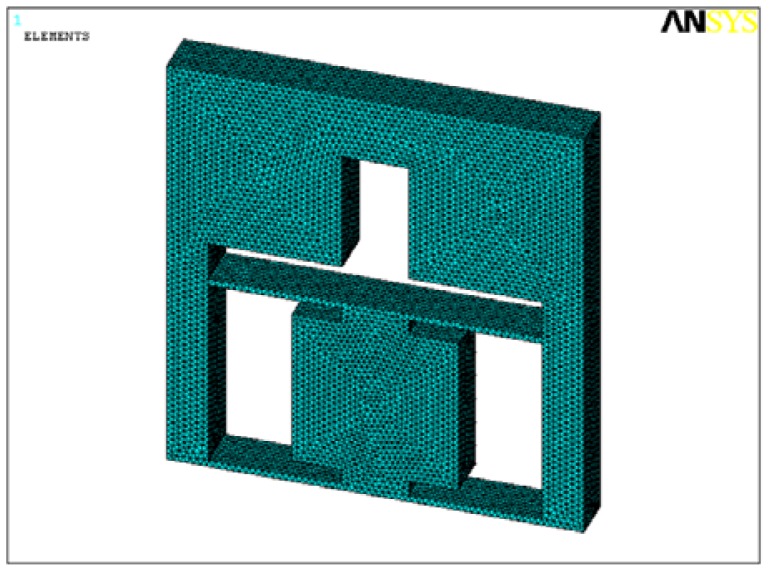
The mesh model of the flexure hinge frame.

In order to make sure that the flexure hinge frame has enough strength and good dynamic performances, static and modal analysis of the structure were carried out. Geometric parameters of the elastic plate are selected as follows. Length *l*_1_ is 40 mm, width *w*_1_ is 6 mm and thickness *t*_1_ is 0.8 mm. Considering that size of strain gauges is so small that they affect the flexure hinge frame very lightly, the analysis model does not include these four strain gauges and the mesh model is shown in Figure 7.

**Figure 8.**
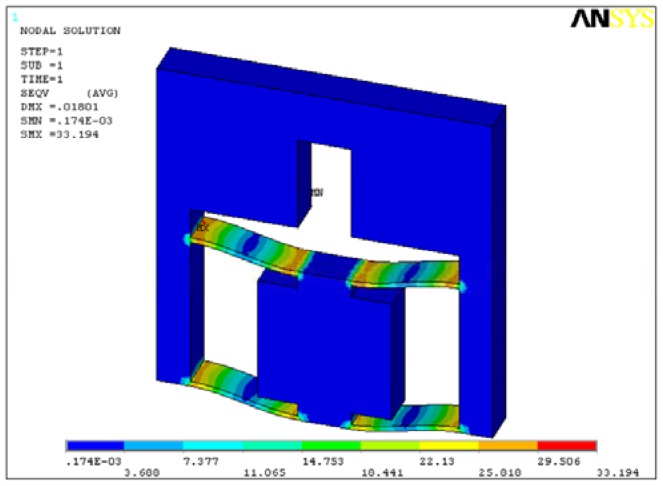
Stress distribution of the flexure hinge frame.

The main purpose of static analysis is to verify the structure strength, so the worst condition that two parallel plates have deformation of 18 μm is applied on the area on which the piezoactuator is installed. The upper surface is fixed. The analysis result is shown in Figure 8.

The maximum stress is 33.19 MPa less than the yield strength of 65 Mn being 432 MPa. Like the analysis results in Section 4.1, larger stress occurs in the root of parallel plates and near the loading area.

**Figure 9.**
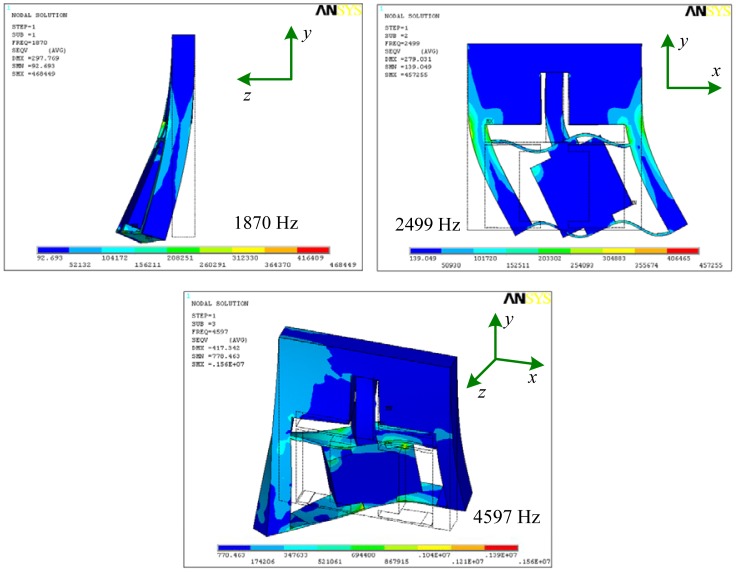
The first three order mode shapes of the precision positioning platform.

Modal analysis is one of the effective methods to examine the dynamic performance of mechanical structures and systems. From modal analysis, natural frequencies and mode shapes which are important parameters when the structure subjects to dynamic loads can be obtained easily. Figure 9 is the first three order mode shapes of the precision positioning platform, corresponding to the first three order natural frequencies of 1,870, 2,499 and 4,597 Hz, respectively. Based on the analysis mentioned above, the structure has a high first order frequency of 1,870 Hz and it can be used at high frequency condition.

## 5. Experiments

**Figure 10.**
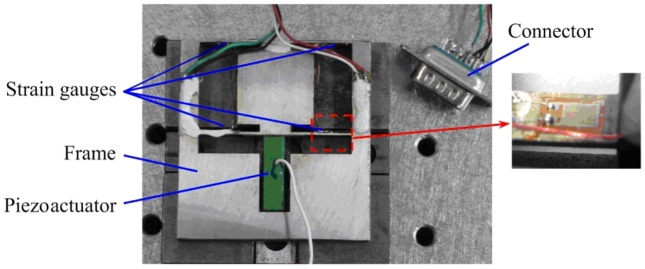
The photograph of the developed precision positioning platform.

**Figure 11.**
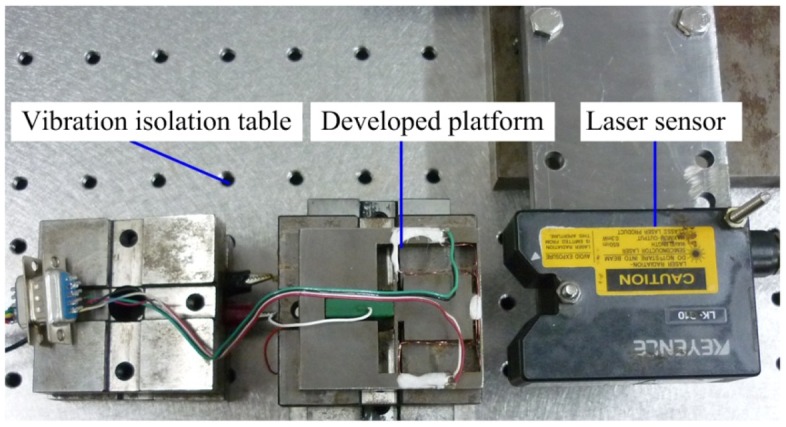
The experimental setup.

Figure 10 is a photograph of the developed precision positioning platform. The calibration experiment was carried out to evaluate the performance of the developed strain gauge sensor. Output performances of the platform were tested via the calibrated strain gauge sensor. The calibration experimental setup is shown in Figure 11. In order to reduce external disturbances on sensing and measurement system, the Newport precision vibration isolation table is used to mount the developed platform. The LK-G10 type laser displacement sensor with resolution of 10 nm is used to calibrate the developed gauge sensor.

### 5.1. Sensor Calibration

**Figure 12.**
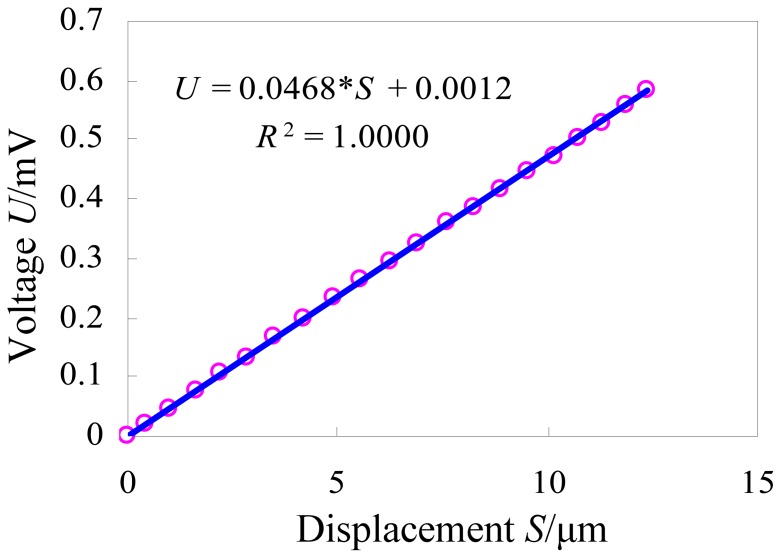
The curve between the output displacement (*S*/μm) and the output voltage (*U*/mV).

The calibration experimental system was established as shown in Figure 11. We adjust the laser displacement sensor to a suitable position so as to obtain the best measuring accuracy. Then we apply a voltage signal to the piezoactuator, and record the output displacement and the output voltage simultaneously. According to the recorded values, the curve between the output displacement (*S*/μm) and the output voltage (*U*/mV) is drawn in Figure 12. The least squares fitting method is used to obtain the relationship between these two sets of data. The slope of the fitted curve demonstrates the sensitivity of the developed strain gauge sensor which is about 0.0468 mV/μm. The linear correlation coefficient *R*^2^ is equal to 1, which indicates that the sensor has good linearity.

### 5.2. Output Performance of the Platform with Open-Loop Control

Considering the stiffness of the flexure hinge frame along driving direction of the piezoactuator, the maximum steady-state output displacement of the precision positioning platform can be given as [3]:

(7) $$S_{\max}=\frac{K_{\text{pzt}}}{K_{\text{pzt}}+K_{\text{fh}}}S_{0}$$

where *S*_max_ is the actual maximum steady-state output displacement of the precision positioning platform. *S*_0_ is the nominal maximum output displacement of the piezoactuator. *K*_pzt_ and *K*_fh_ are the equivalent stiffnesses of the piezoactuator and the flexure hinge frame. The equivalent stiffness of the used piezoactuator is about 48.9 N/μm and the equivalent stiffness of the flexure hinge frame with the structure illustrated in Figure 1 can be obtained by:

(8) $$K_{\text{fh}}=\frac{32Ew_{1}t_{1}^{3}}{{\left(l_{1}-b\right)}^{3}}$$

where *b* is width of the loading area.

According to the Formula (8), the stiffness of the flexure hinge is 0.618 N/μm. Then the maximum steady-state output displacement of the precision positioning platform can be given as:

(9) $$S=\frac{48.9}{48.9+0.618}S_{0}=0.9875S_{0}$$

Corresponding to the maximum applied voltage of 150 V, the nominal maximum output displacement of the piezoactuator is 17.4 ± 2 μm. Usually, the maximum applied voltage does not exceed 100 V during the applications, especially for the nanoindentation application, so corresponding to the maximum applied voltage of 100 V, the theoretical maximum output displacement is in the range of 10.3 μm∼12.9 μm.

**Figure 13.**
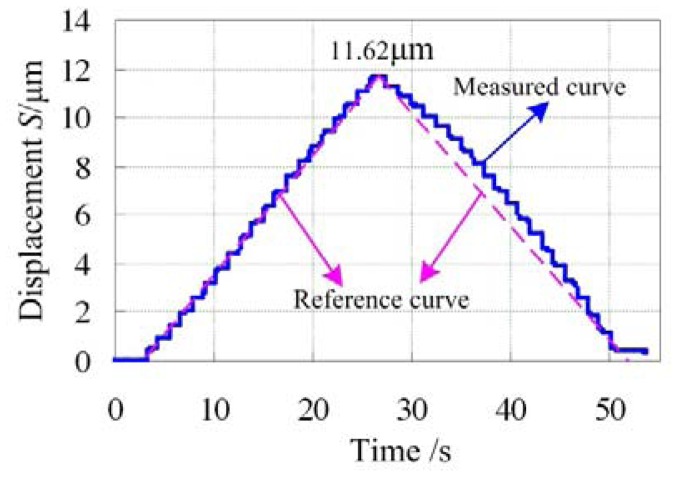
The output curve of the precision positioning platform with open-loop control.

The open-loop control experiment was carried out to test the output performance of the precision positioning platform manually and the results are shown in Figure 13. The step-curve is the measured output curve. It mainly consists of two portions. One is the displacement increasing and the other is the displacement decreasing, each of which has 20 steps corresponding to the voltage range of 0 V∼100 V. The maximum output displacement is about 11.62 μm which is in the range of prediction values, and each step is about 0.581 μm. The dotted line is the reference curve. Comparing the measured curve and the reference curve, it can be concluded that the portion of the displacement increasing is nearly linear but the portion of displacement decreasing expresses obvious nonlinearity, which is mainly caused by the hysteresis of the piezoactuator. In order to solve the problem of nonlinearity, closed-loop control should be developed by the embedded strain gauge sensor.

### 5.3. Output Performance of the Platform with Closed-Loop Control

**Figure 14.**
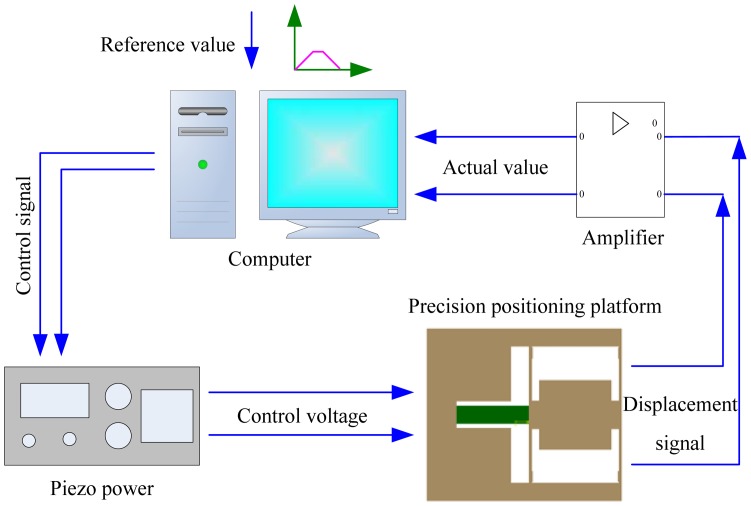
Schematic of the closed-loop control system.

The closed-loop control system is shown in Figure 14. The precision positioning platform is designed to realize precision positioning of the diamond indenter during the indentation. Its compact structure is very suitable for *in situ* nanoindentation inside the scanning electron microscope which requires a compact experimental device. Control signal of the trapezoidal reference curve worked as the displacement control mode during the indentation tests is sent to the piezo power with an internal 16-bit D/A card. Then the control voltage applies to the piezoactuator which will deform and push two parallel elastic plates to output precision displacement. The embedded strain gauge sensor converts the displacement signal to a voltage signal. The voltage signal is filtered and amplified by the amplifier, and then converted to digital signal by an embedded 16-bit A/D card inside the computer. Comparing the reference value and the actual value, the error value is obtained and sent to the piezo power again. The process mentioned above is repeated until the actual value is approximately equal to the reference value.

**Figure 15.**
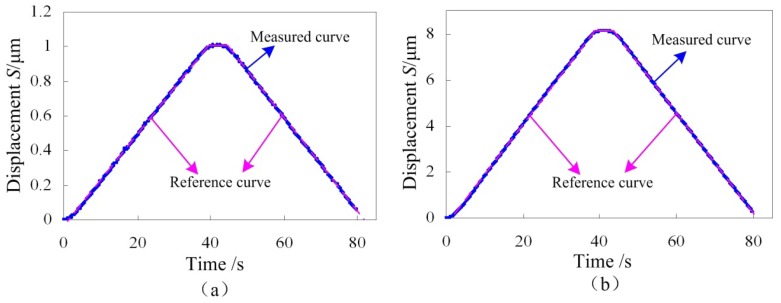
The closed-loop control curves with different maximum output displacement.

The closed-loop control curves are shown in Figure 15. Two different maximum output displacements are selected to verify the feasibility of the control system in different ranges. Comparing the measured curves and the reference curves, both the displacement increasing portion and the decreasing portion have good linearity, demonstrating that the control system is functional, so the designed precision positioning platform integrating strain gauges and a piezoactuator can be used for the *in situ* nanoindentation application, which is the subject of our future work.

## 6. Conclusions

This paper proposes a compact precision positioning platform. Using the embedded strain gauges, the output displacement of the developed platform is measured precisely. The work principle was introduced and the mathematical model of the strain gauge sensor was established. Effects of geometric parameters of two parallel plates on Von Mises stress distribution were studied by the finite element method. Analysis results show that length *l*_1_ and thickness *t*_1_ affect the Von Mises stress of the elastic plate obviously, while width *w*_1_ has smaller effect. The stress in the root of the plate or near the loading area is larger than other places, so strain gauges are suitable for gluing on the root of the plate or near the loading area. Results of static and dynamic analysis of the structure demonstrate that the structure has enough strength, with a high first order frequency of 1,870 Hz.

The calibration experiment of the strain gauge sensor was carried out. The linear correlation coefficient *R*^2^ of the calibration curve is equal to 1, which indicates that the sensor has good linearity. Sensitivity of the developed strain gauge sensor was obtained and it is about 0.0468 mV/μm.

The output performance of the proposed platform with open-loop control was measured by the calibrated strain gauge sensor. Corresponding to the maximum applied voltage of 100 V, the maximum output displacement is about 11.62 μm, agreeing well with the theoretical prediction values. With open-loop control, the portion of displacement increasing expresses good linearity, but obvious nonlinearity is observed during the portion of decreasing displacement.

The closed-loop control system was established to solve the problem of nonlinearity of the platform. Experimental results demonstrate that for the displacement control process, both the displacement increasing portion and the decreasing portion have good linearity, verifying that the control system is functional.

Through analysis and experiments, we can conclude that the proposed compact precision positioning platform was successfully designed. It has a small size but can realize displacement measurements with the embedded strain gauges, which is useful for the closed-loop control and structure miniaturization of piezo devices. It has potential applications in nanoindentation and nanoscratch tests, especially in field of *in situ* nanomechanical tests which require compact structures.
